# Identifying Subgroups of Patients With Chronic Nonspecific Low Back Pain Based on a Multifactorial Approach: Protocol For a Prospective Study

**DOI:** 10.2196/resprot.9224

**Published:** 2018-04-23

**Authors:** Kevin Rose-Dulcina, Nicolas Vuillerme, Anne Tabard-Fougère, Romain Dayer, Dennis E Dominguez, Stephane Armand, Stéphane Genevay

**Affiliations:** ^1^ Willy Taillard Laboratory of Kinesiology Department of Surgery Geneva University Hospitals and University of Geneva Geneva Switzerland; ^2^ Autonomie, gérontologie, e-santé, imagerie & société Laboratory Department of Chemistry, Biology and Health University Grenoble Alpes Grenoble France; ^3^ LAI Jean-Raoul Scherrer University of Geneva and University Grenoble Alpes Grenoble France; ^4^ Institut Universitaire de France Paris France; ^5^ Division of Paediatric Orthopaedics Faculty of Medicine Geneva University Hospitals Geneva Switzerland; ^6^ Division of Orthopaedic and Trauma Surgery Faculty of Medicine Geneva University Hospitals Geneva Switzerland; ^7^ Division of Rheumatology Faculty of Medicine Geneva University Hospitals Geneva Switzerland

**Keywords:** low back pain, chronic pain, activities of daily living, psychology, electromyography, biomechanical phenomena, classification

## Abstract

**Background:**

Low back pain, especially nonspecific chronic low back pain (LBP), the leading cause of disability worldwide, represents both social and economic problems. Different therapeutic management techniques can be used, but their effects vary. Clinicians and researchers attribute the variation in the efficacy of therapeutic and management techniques to the heterogeneity of the nonspecific chronic low back pain population, and they agree that nonspecific chronic LBP must be subgrouped.

**Objective:**

This study aims to identify nonspecific chronic LBP subgroups based on a multifactorial approach, including biomechanical, physical, and psychosocial data.

**Methods:**

A total of 100 nonspecific chronic LBP patients and 30 healthy participants aged between 18 and 60 years will be recruited for this prospective study. A psychosocial profile will be established using questionnaires on anxiety, depression, functional disability, pain, fear of pain, avoidance belief, and physical activity. A physical capacity evaluation will be conducted. It will evaluate flexibility of the hips, lumbar spine, and lateral thoracolumbar segment, as well as trunk (extensor and flexor) muscle endurance. The subjects will perform functional daily life activities, such as walking, object lifting, forward bending, sit-to-stand, stand-to-sit, balance, and usual postures. Full body kinematics, kinetics, and surface electromyography of the trunk and hip muscles will be assessed during these tasks. The clustering classification methods for the statistical analysis will be determined according to the data and will be used to identify the subgroups of nonspecific chronic LBP patients.

**Results:**

Data collection started in September 2017 and will be completed with the inclusion of all the participants (100 nonspecific chronic LBP and 30 control). The study results will be published in peer-reviewed journals and presented at relevant international conferences.

**Conclusions:**

Numerous studies have showed that the therapeutic management of nonspecific chronic LBP is difficult and has inconstant effects caused by the complexity and heterogeneity of nonspecific chronic LBP. Identifying subgroups with a multifactorial approach is more comprehensive and closer to the pathophysiology of nonspecific chronic LBP. It also represents benefit interests and a challenge both clinically and socially. The perspective of this study is expected to support clinicians for a more adapted therapeutic management for each subgroup.

## Introduction

### Background

Low back pain (LBP) has been the leading cause of disability worldwide since 1990 [1] and has a lifetime prevalence of 84% in industrialized countries [2]. LBP is defined as pain and discomfort of varying duration. It is localized below the costal margin and above the inferior gluteal folds, with or without irradiation in the lower limb [3]. LBP is considered chronic when pain duration exceeds 3 months [4,5] and accounts for 10% of the cases and represents 70% to 90% of the total LBP cost [6]. A recent study reported that chronic LBP treatment in the United States costs between US $85 and US $238 billion annually [7]. In Switzerland, chronic LBP costs between 1.6 and 2.3 of the gross domestic product [8]. In France, chronic LBP is one of the costliest diseases with 6-month direct costs of US $ 884,85 per patient [9]. Furthermore, the World Health Organization recently reported that chronic LBP is one of the major causes of professional health-related absences [10]. Therefore, chronic LBP represents a significant worldwide problem with major medical, social, and economic impact.

Knowledge on the LBP pathophysiology is not sufficient. A precise diagnosis can only be obtained in 10%-15% of the cases [11,12]. Therefore, LBP is mostly categorized as nonspecific. Nonspecific LBP is a constellation of symptoms not attributable to a known specific pathology (ie, infection, tumor, osteoporosis, fracture, structural deformity, inflammatory disorder [eg, ankylosing spondylitis], radicular syndrome, and cauda equina syndrome) [2,11,13,14]. In addition, pathologies that are known as possible causes of pain (eg, osteoarthritis, disc disease, or cracked discs) do not explain the onset of symptoms on their own due to a similar prevalence of these pathologies being found in asymptomatic subjects [2,15].

Nonspecific chronic LBP results from a variety of factors which can interact with each other. These include biomechanical, psychosocial, physical, environmental, genetic, and cultural factors [2]. The diversity of these factors and the complexity of their interactions could explain the difficulty in establishing a specific etiology of nonspecific chronic LBP. In the absence of a clear diagnosis, physicians face a therapeutic challenge caused by the large number of available treatments (eg, drugs, physiotherapy, physical exercise), for which the overall effect is small to moderate [16]. The poor efficiency of the available treatments is attributed to the heterogeneity of nonspecific chronic LBP patients [17]. Therefore, identifying nonspecific chronic LBP patient subgroups is essential [18] and will help optimize therapeutic management [19-22]. The need for nonspecific chronic LBP patient subgroups was highlighted by 84% of primary care clinicians on a large-scale survey [18].

### Prior Work

Numerous differences between nonspecific chronic LBP patients and healthy subjects were reported using various clinical features. Regarding genetic factors, some studies have reported that genes result in a predisposition to intervertebral disc degeneration [23] or can alter pain perception [24]. Psychological factors, such as pain catastrophyzing, are altered in nonspecific chronic LBP patients and can influence physical performance [25]. In terms of physical capacities, the nonspecific chronic LBP population presented with reduced endurance and higher fatigability of the trunk extensor muscles [26] and lower hip and lumbar flexibility, correlating with nonspecific chronic LBP severity [27]. With regards to biomechanical factors, nonspecific chronic LBP patients exhibited kinematic and muscle activity impairments [28,29]. When compared to healthy subjects, nonspecific chronic LBP patients showed decreased pelvis rotation during gait [29] and an increased stiffness of the spino-pelvic complex [30]. Moreover, nonspecific chronic LBP patients presented with decreased maximum range of motion and velocity between the lumbar spine and the hips during the sit-to-stand (STS) task [31] associated with stiffer spine movements [32]. Meanwhile, during the lifting task, they used different kinematic strategies, especially in lift speed and hip and knee flexion [33] and presented with less variability in kinematic patterns [34]. Alterations were also found in the trunk and hip muscle surface electromyography (sEMG). Nonspecific chronic LBP subjects presented with higher global trunk muscle activity during gait [35] or lifting tasks [36]. Many studies highlighted an exacerbated lumbar erector spinae activity (absence of the flexion-relaxation phenomenon) at full trunk forward flexion [37-39].

Nonspecific chronic LBP patient subgroups have previously been identified based on biomechanical parameters. Slaboda et al [40] identified 2 subgroups based on lift kinematic patterns, whereas Dankearts et al [20] discriminated 2 subgroups based on sitting posture. They also discriminated the subgroups on trunk muscle activity, posture, and movement [20,41], which make the biomechanical analysis of nonspecific chronic LBP patients relevant for a better understanding of this pathology and could help to discriminate different subgroups.

However, identifying subgroups only from a biomechanical analysis is not comprehensive enough due to the emotional and behavioral consequences of pain, which contributes to the persistence of pain and treatment outcomes, and due to the multi-factorial features of nonspecific chronic LBP [42]. Anxiety and depression play a major role in pain chronicity in nonspecific chronic LBP patients [43]. Psychosocial parameters have also been observed to influence kinematic and muscle activities. Indeed, a high level of pain catastrophizing was associated with a decrease in the activation time of the spinal muscle (multifidus) in LBP patients during forward bending [44] and a lower performance time in the trunk extensor endurance test [25]. Lamoth et al [45] showed that the fear of pain altered muscle activity during gait, with a decrease of the erector spinae sEMG mean amplitude. Thus, the identification of the nonspecific chronic LBP subgroups should be based on the multifactorial parameters (ie, biomechanical, physical, and psychosocial data) linked to nonspecific chronic LBP.

### Aim

This study aims to identify the subgroups of nonspecific chronic LBP patients based on a multifactorial approach, including biomechanical, physical, and psychosocial data.

## Methods

### Study Design

This is a prospective study approved by the Research Ethic Cantonal Commission of the University Hospitals of Geneva (HUG) with reference CER: 14-126. All study data and human material will be handled confidentially and coded with a unique study number. Only the research team will have access to the data.

### Participants

The study population consists of 18- to 60-year old adults from the Geneva area and is divided into 2 groups, namely patients suffering from nonspecific chronic LBP (LBP group) and healthy participants (control group). Both groups will be evaluated in the Willy Taillard Laboratory of Kinesiology of the HUG. Patients will be recruited from the Division of Rheumatology and the Division of Orthopaedic and Trauma Surgery of the HUG.

The patient inclusion criteria are as follows; (1) suffering from nonspecific chronic LBP, (2) duration of at least 3 months, (3) pain intensity over 3/10 on a visual analogical scale, (4) aged between 18 and 60 years, (5) no pain on other parts of the body (except irradiation of nonspecific chronic LBP), and (6) no specific pathology such as infection, tumor, osteoporosis, fracture, structural deformity, inflammatory disorder (eg, ankylosing spondylitis), radicular syndrome, and cauda equina syndrome. The healthy participant inclusion criteria are as follows; (1) aged between 18 and 60 years, (2) no back pain for at least 6 months, and (3) no pain in any part of the body.

The subjects who present with a history of back surgery, a body mass index over 30 kg/m^2^, inability to understand French, and pregnancy will be excluded from both groups. All participants included in our study will provide written informed consent to participate.

### Sample Size

The sample size calculation was computed using GPower software (Heinrich Heine University, Dusseldorf, Germany) [46]. This calculation was based on previous studies which identified 2 nonspecific chronic LBP subgroups from sEMG and posture variables. Dankaerts et al [20] found greater lumbar multifidus activity during slumped sitting among the control group (n=34), pooled nonspecific chronic LBP group (n=33), and within 2 subgroups of nonspecific chronic LBP (n=20 and n=13). Meanwhile, Astfalck et al [47] found a difference between the upper lumbar angle in the sitting posture of the control group (n=28) and the 2 nonspecific chronic LBP subgroups (n=13 and n=15). The number of participants per group should be between 17 to 21 for comparison with healthy participants and between 27 to 32 for each nonspecific chronic LBP subgroup for differentiation between 2 and 3 subgroups with a statistic power up to 80% and a 5% alpha error. Therefore, we will include 100 nonspecific chronic LBP patients and 30 healthy participants.

### Data Collection

#### Task Description

The International Classification of Functioning (ICF) defines the typical spectrum of problems in the functioning of patients with LBP and highlights the main areas and functions of interest in the study of LBP [48]. On the basis of the short version of ICF [49], the physical capacities of the patient will be assessed by assessing the flexibility of the hips in flexion and extension, the lumbar spine in flexion, the thoracolumbar segment in lateral flexion, and the trunk extensor and flexor muscle endurance. Functional abilities will be assessed from daily life activities such as gait, object lifting, forward bending, STS (and the reverse), balance, and usual posture (standing and sitting). Kinematics, kinetics, and sEMG will be assessed during the execution of these functional tasks.

#### Trunk Muscle Endurance

The Sorensen test, which is considered as the gold standard for this measure [50], will be performed to determine trunk extensor endurance [51,52]. The participants will lie on the examining table in a prone position with the upper edge of their iliac crests' aligned along the edge of the table. The lower body will be fixed to the table by 3 straps located at the level of the pelvis, knees, and ankles. Meanwhile, the Shirado test will be performed to determine trunk flexor endurance [53]. The participants will lie on the examining table in a supine position and will raise their lower extremities until their scapulas' are off the table with a 90° flexion of the hip and knee joints. These tests are considered valid, safe, reliable, and easy to perform in participants with and without nonspecific chronic LBP [54,55]. Participants’ arms are folded across the chest for the duration of both trunk muscle endurance tests. Note that the participants will be asked to hold the original positions for as long as possible, but not exceeding a 240 s time limit. A 15 min rest is allowed between the two endurance tests.

#### Trunk and Hip Flexibility

The hip and trunk muscles flexibility in the nonspecific chronic LBP population will be evaluated using the straight leg raise test, the Thomas test, and the finger-tip-to-thigh test. These are valid, reliable, and largely used tests. The tests will assess hamstring flexibility [17,56-58], hip flexor flexibility (psoas-iliacus and rectus femoris) [59-62], and measure the lateral trunk range of motion [63,64]. All flexibility tests will be performed according to the methods set out by Norkin and White [65].

#### Balance

Participants’ balance in standing and sitting postures will be evaluated. For the sitting condition which limits the influence of the lower limb, the participants will be seated on an adjustable stool with the middle of the thighs on the edge of the stool and with their feet dangling. For the standing posture, the participants will stand with 10 cm between their heels and a self-selected angle between the feet [66]. For both postures, the participants must make sure their trunk is erect, fix their head in a neutral position, look ahead, keep arms along the trunk, and move as little as possible. The participants will stand on a force plate and their balance will be assessed under 4 conditions with 3 repeated trials of 30 s per condition. The conditions under which the balance of the participant will be assessed are eyes closed or opened, with stable or unstable support. To create the unstable support an Airex balance pad (50 cm length × 41 cm width × 6 cm thickness) will be used. The condition with the eyes closed are used to avoid visual compensations [67] and unstable conditions are used to challenge the participants’ balance [68].

#### Usual Postures

The usual sitting and standing postures of each participant will be evaluated. For the sitting posture evaluation, an adjustable stool will be placed on a force plate and the participants will be asked to be seated in a self-selected position with their feet on another force plate. The stool height will be adjusted for each participant to fix the hip and knee flexion at 90°. For the usual standing posture evaluation, the participants will be asked to stand in a self-selected upright position with both feet on the same force plate. For both these usual static postures, the participants will look ahead, and the kinematic will be recorded for 10 s in the posture.

#### Gait

The participants will be asked to walk barefoot at 3 different speeds (ie, self-selected, fast, and slow) along a 10 m walkway to assess their gait. Data will be collected for at least 10 gait cycles for each participant and the speed will be monitored.

#### Lifting Task

Two lifting tasks will be performed. For both tasks, the participants will start on a force plate in an upright standing position, bend down to lift a box and return to an upright standing position with 90° flexion of elbows holding the box. They will maintain this posture for 4 s, and then bend down to place the box to the ground before returning to the initial posture. This test will be performed under two conditions. The first condition is a usual lift, where the participants are asked to lift the box with a self-selected strategy, and no more instruction will be given [69]. The second condition is a standardized lift based on deadlift methods [70]. This will be used to compare the muscle strategies for the same movement between the subjects. The usual lift will be performed before the standardized lift to avoid behavioral adaptations.

Three trials will be performed per condition with 2 min rest between each condition. The weight of the box will be adjusted to 10% of the participant’s weight for each condition. The participant will be instructed to stay on the force plate for the duration of the test.

#### Trunk Forward Bending

The participants will start standing in an upright position (standing phase), flex the trunk as far forward as possible with their knees extended (flexion phase), maintain this trunk full-flexion position (full flexion phase), and then return to an upright standing position (extension phase). Each phase will last for 4 s, and an audible metronome will be used to regulate the movement timing. Three trials will be performed, and only the second trial will be used for analysis [71].

#### Sit-to-Stand

The STS tasks will be performed under the following three conditions: (1) usual STS, (2) standardized STS, and (3) 5 consecutive STSs. In the usual condition, the participants will sit in a self-selected position on a stool placed on a force plate with their feet placed on another force plate. No more instructions on posture will be given for this condition. In the standardized STS, the participants will be barefoot and asked to sit upright on an adjustable stool with their trunk straight and arms crossed on the chest. The stool will be placed on a force plate, the participant’s feet will be placed on a second force plate, and the stool height will be adjusted for each participant to fix the hip and knee flexion at 90° in the starting position.

For both the usual STS and standardized STS, the participants will stand up after 4 s of sitting, maintain the upright standing position with knees fully extended for 4 s, return to the initial sitting position, and maintain it for 4 s. Three trials will be performed for both the STS and standardized STS tasks.

The 5 consecutive STS task provides information on the global capacity of the participant to perform the STS task. The participants will have the same start position as the standardized STS task. The participants will then be asked to perform 5 consecutive STS movements as fast as possible. As a precautionary measure, an investigator will stand near the participant to prevent possible falls. To evaluate the total task duration, the start and end points of the task will be defined by the mean value of the anterior-posterior center of the pressure displacement during the usual sitting phase before and after the task was completed [72].

#### Psychosocial Profile

The psychosocial profile will be explored using patient-reported outcomes to evaluatef anxiety, depression, functional disability, fear of pain, avoidance belief, and physical activity (PA). All the questionnaires will be self-completed before the experiments, except for the PA questionnaire which will be completed by the investigator with the participant during the course of the experiments.

#### Anxiety and Depression

Anxiety and depression are parameters which play an important role in the sustainability of pain; hence, they are factors of pain chronicity [42]. The Hospital Anxiety and Depression Scale (HADS) [73] is widely used to evaluate mental disorders [74] in the LBP population [75]. This study will use the French version of the HADS introduced by Lépine et al [76], which has been used in other studies conducted on French-speaking populations [77-79].

#### Functional Capacity

The functional capacity evaluation is recommended when studying LBP [80]. Functional capacity is indeed an interesting parameter to evaluate the interference of pain on daily life [81]. One of the most used and recognized assessment tools is the Oswestry Disability Questionnaire (ODI) [82], which is specific for LBP [83]. The French validated version of the ODI [84] will be used for this study.

#### Pain Catastrophizing

A systematic review shows that pain catastrophizing can predict the degree of pain, disability, and mediated treatment efficacy in the nonspecific chronic LBP population [85]. The pain catastrophizing scale (PCS) was introduced by Sullivan et al [86], and his validated French version [87] will be used for this study.

#### Fear and Belief

Fear avoidance beliefs are reported to be factors for the delayed recovery and chronicity of pain in the nonspecific chronic LBP population [88]. One of the questionnaires used to identify fear avoidance beliefs is the Fear Avoidance Beliefs Questionnaire (FABQ) [89]. This study will use the French version of the FABQ validated by Chaory et al [90].

#### Physical Activity

PA plays an important role in the prevention of nonspecific chronic LBP. The participant’s weekly PA will be assessed using the Global Physical Activity Questionnaire (GPAQ) developed by the World Health Organization [91]. This questionnaire has already been used in studies on the nonspecific chronic LBP population [92].

#### Pain

Pain is a key symptom in nonspecific chronic LBP, therefore, its evaluation during the course of the study is recommended [49,93]. The intensities of current pain, pain in the last 24 h, pain in the last week, pain in the last month, and pain in the last 3 months will be quantified with a visual analogue scale largely used in the nonspecific chronic LBP population [29,94-96].

### Materials and Parameters

#### Electromyographic Activity

The sEMG will be bilaterally collected from 3 back muscles (ie, lumbar multifidus, iliocostalis lumborum, and lumbar erector spinae), 2 abdominal muscles (ie, transverse fibers of the abdominal external oblique and rectus abdominus), gluteus medius, semitendinosus, and the rectus femoris muscle. Moreover, 16 active surface electrodes (model: Trigno, Delsys Inc, Boston, MA, USA) will be used to collect the sEMG signals at a sampling frequency of 1000 Hz. The skin at the electrode sites will be shaved, abraded, and cleaned with alcohol prior to measurement. The electrodes will then be positioned relative to the muscle fiber direction, following the surface EMG for noninvasive assessment of muscles project recommendations [97].

The sEMG activation pattern, time of cocontraction (TCC), and cocontraction index (CCI) [98] will be calculated for gait, STS, and lift tasks. The TCC is the time for simultaneous activation of a pair of muscle groups over a specified number of data points (activation thresholdbaseline+3 SD; duration activity threshold: 5 ms) [99]. The CCI is defined as the degree of coactivation for a pair of muscle groups over a specified number of data points. The TTC and CCI will characterize muscle coordination. The flexion-relaxation ratio will be calculated and used to detect and quantify exacerbated back muscle activity for trunk forward bending [71]. The muscle fatigability of the back and abdomen muscles will be evaluated using the sEMG median frequency evolution during endurance tasks [100].

#### Kinematics

The kinematic parameters will be recorded using a 12-camera motion analysis system (Oqus7+, Qualisys, Göteborg, Sweden) set at a sampling frequency of 100 Hz. The participants will have 35 reflective markers (14 mm diameter) placed on the skin at defined anatomical and technical landmarks on the head, trunk, and pelvis and bilaterally on the arms, thighs, shanks, and feet according to the full-body Plug-in-Gait model [101]. Additional markers will be placed on the spinous process of T2, T4, T6, T8, L1, L3, L5, and S1 to assess the sagittal plane curve of the spine [102,103].

The thorax, lumbar, pelvis, hip, knee, and ankle kinematics (maximum angle, range of motion, and speed) will be calculated in 3 planes for all tasks. The lumbar/hip ratio will be calculated for the trunk forward bending, STS, and lift tasks [104,105]. The relative phase between the pelvis and the thorax segment and the spatiotemporal parameters (ie, walking speed, cadence, stance phase, and step length) will be calculated during gait [106]. The thorax movement during the balance tasks will characterize the trunk sway [107].

#### Kinetics

Two force plates (AMTI Accugait, Watertown, NY, USA) at a sampling frequency of 1000 Hz will be used to measure the ground reaction forces. The center of pressure displacement (range and speed) will be calculated for the balance tasks to assess the balance capacity [68,108,109]. All kinetic, kinematic, and sEMG data will be synchronized together.

### Experimental Procedure

**Figure 1:**
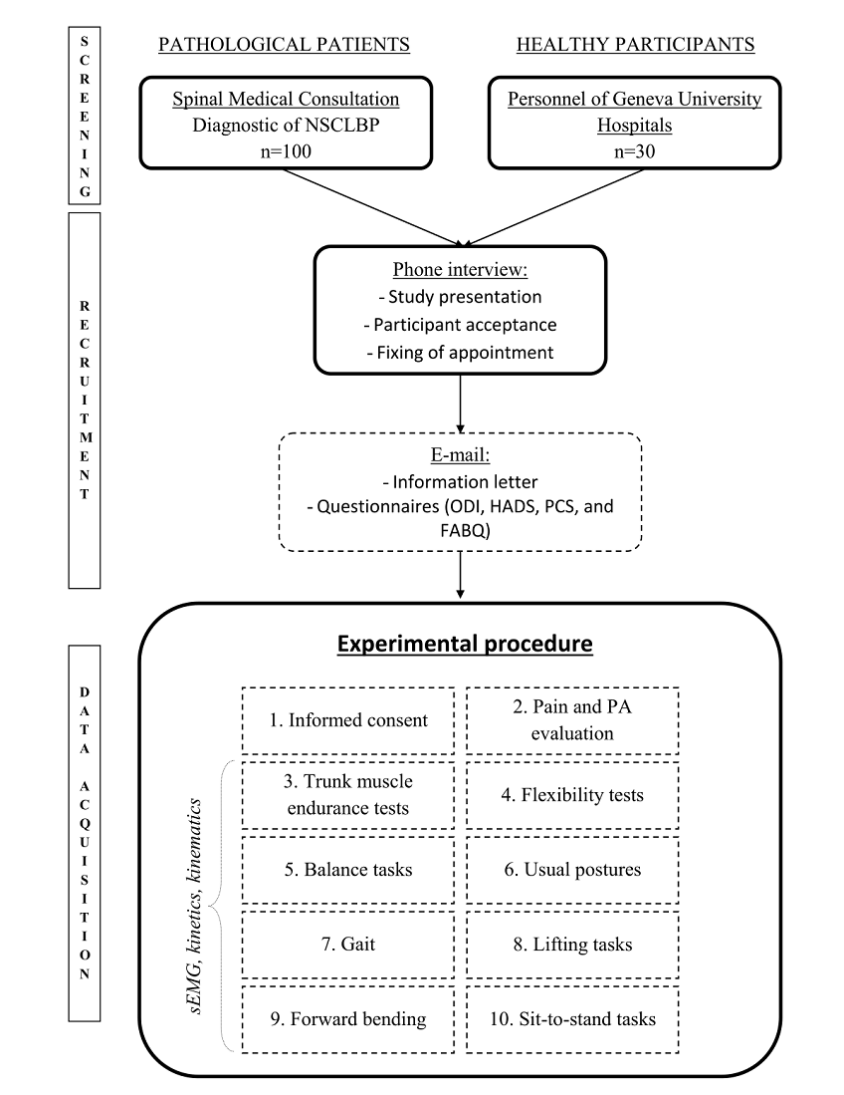
Flow diagram of the study. FABQ: fear-avoidance belief questionnaire; HADS: Hospital Anxiety and Depression Scale; NSCLBP: nonspecific chronic low back pain; ODI: Oswestry Disability Index; PA: physical activity; PCS: pain catastrophizing scale; sEMG: surface electromyography.

To introduce the study, a phone interview will be conducted by the investigator after nonspecific chronic LBP is diagnosed by a spinal consultant. An information letter will be sent to the patient (by email or post) once he/she agrees to voluntarily participate in this study. An appointment time will then be scheduled. Upon arrival, the participants will complete the HADS, ODI, and PCS questionnaires. The GPAQ and Pain Evaluation will be completed by the investigator during the interview with the participant. All sEMG sensors will be placed after skin preparation as outlined above. The participants will then perform the flexor endurance, extensor endurance, and flexibility tests. Next, the reflective markers will be placed, and the participants will perform the functional tasks in the order listed above with a minimum rest period of 3 min between each task. A pain assessment will be made after each task to quantify the pain generated by the task, using current pain as a reference. The total duration of this protocol (Figure 1) will be 120 min per participant.

### Data Analysis

The joint kinematics and kinetics data will be computed using Visual3D (C-Motion, Inc, Germantown, MD, USA). Data extraction will be performed using MATLAB R2015b (MathWorks, USA) and the open-source Biomechanical ToolKit package for MATLAB [110]. R software v.3.1.3 will be used for all statistical analyses. Data will be reduced with principal component analysis. Meanwhile, *K*-mean or descending hierarchical clustering classification methods will be used to identify the nonspecific chronic LBP subgroups. The clustering classification methods will be determined according to the data. In addition, a statistical inference test (parametric or nonparametric depending on the normality of the data distribution) will be applied to compare the nonspecific chronic LBP patients with healthy participants and to compare the different patient subgroups (*P*<.05).

## Results

The data collection started in September 2017 and will be completed with the inclusion of all the participants (100 nonspecific chronic LBP patients and 30 controls). The study results will be published in peer-reviewed journals and presented at relevant international conferences.

## Discussion

### Principal Consideration

This study presents originality and the opportunity to connect large amounts of data about different features of various conditions with the same population sample. The results should allow for a better understanding of nonspecific LBP. The perspective of this study is expected to support clinicians for more adapted therapeutic management for each subgroup. Furthermore, this study could provide a reference protocol for functional tasks when nonspecific chronic LBP is studied.

### Limitations

A limitation of this study could include missing data from the participant and/or to the materials used in the study. For example, a nonspecific chronic LBP patient may not be able to perform all the tasks required because of their functional capacity or pain level. An example of missing data from the study material could include the fact that surface EMG may contain artifacts that alter analysis of the muscle activity. Moreover, patients will be recruited from the Orthopedic and Rheumatology service of Geneva University Hospital, which limits generalization of the results to the global nonspecific chronic LBP population. Finally, because previous studies have found two nonspecific chronic LBP subgroups, three subgroups were used for the sample size calculation to ensure that at least two subgroups could be found, but more groups may be found in the clustering analysis.

### Conclusions

Therapeutic management of nonspecific chronic LBP is rather difficult and has inconstant effects because of the complexity of nonspecific chronic LBP and the heterogeneity of nonspecific chronic LBP patients. Identifying subgroups in the nonspecific chronic LBP population represents benefit interests and a challenge both clinically and socially. This study aims to identify subgroups in nonspecific chronic LBP participants which include biomechanical, physical, and psychosocial factors to enhance the targeted therapy.

## Abbreviations

5CSTS: 5 consecutive sit-to-stand
CCI: cocontraction index
FABQ: fear-avoidance belief questionnaire
GPAQ: global physical activity questionnaire
HADS: Hospital Anxiety and Depression Scale
HUG: Geneva University Hospital
ICF: International Classification of Functioning, Disability, and Health
LBP: low back pain
ODI: Oswestry Disability Index
PA: physical activity
PCS: pain catastrophizing scale
STS: sit-to-stand
sEMG: surface electromyography
TCC: time of cocontraction
